# Neuron loss in the 5XFAD mouse model of Alzheimer’s disease correlates with intraneuronal Aβ_42_ accumulation and Caspase-3 activation

**DOI:** 10.1186/1750-1326-8-2

**Published:** 2013-01-14

**Authors:** William A Eimer, Robert Vassar

**Affiliations:** 1 Department of Cell and Molecular Biology, Feinberg School of Medicine, Northwestern University, 303 East Chicago Avenue, Chicago, IL, 60611, USA

**Keywords:** Intraneuronal Aβ_42_, 5XFAD, Alzheimer’s disease, Amyloid-β, Caspase-3, Neuron loss, Apoptosis

## Abstract

**Background:**

Although the mechanism of neuron loss in Alzheimer’s disease (AD) is enigmatic, it is associated with cerebral accumulation of Aβ_42_. The 5XFAD mouse model of amyloid deposition expresses five familial AD (FAD) mutations that are additive in driving Aβ_42_ overproduction. 5XFAD mice exhibit intraneuronal Aβ_42_ accumulation at 1.5 months, amyloid deposition at 2 months, and memory deficits by 4 months of age.

**Results:**

Here, we demonstrate by unbiased stereology that statistically significant neuron loss occurs by 9 months of age in 5XFAD mice. We validated two Aβ_42_-selective antibodies by immunostaining 5XFAD; BACE1^−/−^ bigenic brain sections and then used these antibodies to show that intraneuronal Aβ_42_ and amyloid deposition develop in the same regions where neuron loss is observed in 5XFAD brain. In 5XFAD neuronal soma, intraneuronal Aβ_42_ accumulates in puncta that co-label for Transferrin receptor and LAMP-1, indicating endosomal and lysosomal localization, respectively. In addition, in young 5XFAD brains, we observed activated Caspase-3 in the soma and proximal dendrites of intraneuronal Aβ_42_-labeled neurons. In older 5XFAD brains, we found activated Caspase-3-positive punctate accumulations that co-localize with the neuronal marker class III β-tubulin, suggesting neuron loss by apoptosis.

**Conclusions:**

Together, our results indicate a temporal sequence of intraneuronal Aβ_42_ accumulation, Caspase-3 activation, and neuron loss that implies a potential apoptotic mechanism of neuron death in the 5XFAD mouse.

## Background

The histopathology of Alzheimer’s disease (AD) is characterized by two hallmark lesions, extracellular amyloid-β plaques made of the Aβ peptide, and intracellular neurofibrillary tangles composed of hyperphosphorylated tau (reviewed in [1-5]). In addition to the presence of plaques and tangles in the brain, considerable neuron loss is also a cardinal feature of AD, but the mechanisms of neural cell death are unclear. Importantly, familial AD mutations (FAD) in the genes for amyloid precursor protein (APP), presenilin 1 (PS1), and presenilin 2 (PS2) that cause AD implicate Aβ as an initiating factor in AD pathogenesis (reviewed in [5,6]). These FAD mutations increase the production of Aβ_42_, the 42-amino acid form of the peptide, from APP, which is sequentially cleaved by the β- and γ-secretase enzymes to release the peptide. These results, among others, strongly suggest that Aβ_42_ plays a central early role in the pathophysiology of AD that ultimately leads to the neuron loss and dementia observed in the disorder.

The mechanism by which Aβ_42_ exerts neurotoxicity is poorly understood; many mechanisms have been hypothesized, but none have been definitively proven. The accumulation of intraneuronal Aβ_42_ has been observed in the brains of AD patients and APP transgenic mice, and studies suggest that intraneuronal Aβ_42_ plays a role in neurodegenerative processes relevant to AD (reviewed in [7-9]). Frank neuron loss has been observed in two aggressive amyloid plaque transgenic mouse models that also exhibit accumulation of intraneuronal Aβ_42_ prior to plaque formation: the 5XFAD and APP^SL^PS1K1 lines [10,11]. These transgenic models express multiple FAD mutations that additively increase Aβ_42_ production. In the case of the 5XFAD model, the mouse overexpresses APP with K670N/M671L (Swedish mutation [12]), I716V (Florida mutation [13]), and V717I (London mutation [14]), and PS1 with M146L and L286V mutations [15]. Individually, each FAD mutation enhances Aβ_42_ generation, but together they act synergistically in the transgenic mouse to predominantly make Aβ_42._ Consequently, 5XFAD mice represent a very aggressive amyloid deposition model that develops intraneuronal Aβ_42_ at 1.5 months, plaques at 2 months, memory deficits at 4 months, and neuron loss at 9 months of age [10]. These characteristics make 5XFAD mice a robust model for investigating the role of intraneuronal Aβ_42_ in neuron loss.

Here, we have examined the process of neuronal death in 5XFAD mice and found a correlation between intraneuronal Aβ_42_, neuron loss, and Caspase 3 activation in large pyramidal neurons of the brain. These results suggest a potential role for an apoptotic mechanism in intraneuronal Aβ_42_-mediated neuron loss, and may have relevance for neuronal death in AD.

## Results

### 5XFAD mice exhibit progressive neuron loss in cortical Layer 5 and subiculum

**Figure 1:**
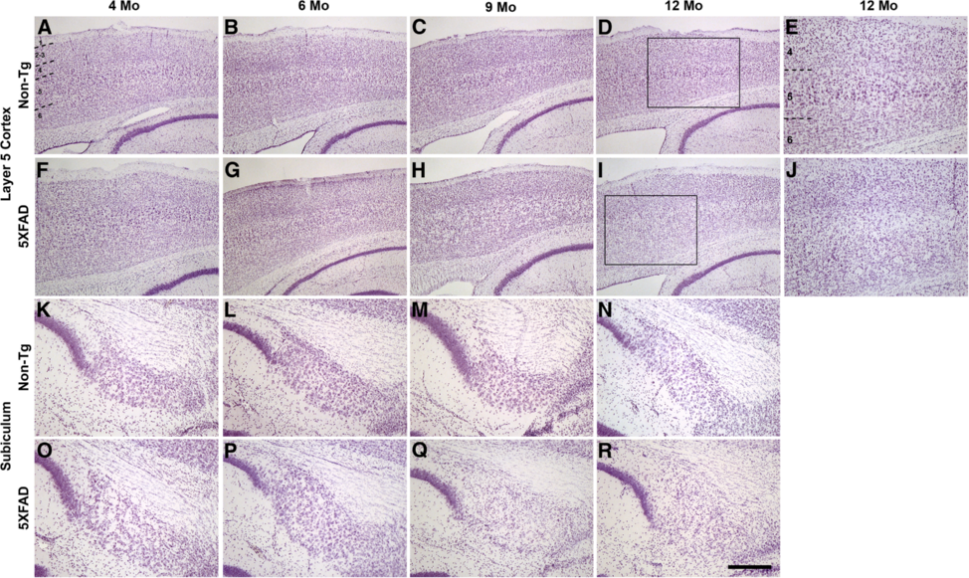
**5XFAD mice exhibit neuron loss in Layer 5 cortex and subiculum.** Parasagittal brain sections from representative 4- to 12-month-old non-transgenic littermate **(A-E, K-N)** and 5XFAD **(F-J, O-R)** female mice were stained with cresyl violet and micrographed to image Layer 5 cortex **(A-J)** and subiculum **(K-R)**. Numbers indicate cortical layers and dashed lines identify borders between layers. Boxes in **D** and **I** outline the areas of increased magnification shown in **E** and **J**, respectively. The 5XFAD mouse exhibits visible loss of large pyramidal neurons at 9 months of age as evident by a decrease in stained neurons **(H, Q, I, R, J)**. Increased magnification **(J)** shows a clear loss of large pyramidal neurons in Layer 5 of the 5XFAD mouse at 12 months compared to the non-transgenic (**E**). Scale bar in **R** = 280 μm for **A-D, F-I**; 110 μm for **E, J**; 80 μm for **K-R**.

The 5XFAD transgenic mouse is one of the few amyloid animal models that exhibits significant neuron loss. Our previous work demonstrated a qualitative reduction of 5XFAD pyramidal neurons in cortical Layer 5 and subiculum at 9 months of age [10]. Moreover, Jawhar and colleagues have shown a significant quantitative decrease of 5XFAD Layer 5 neurons at 12 months of age [10,16]. To extend these findings and determine in greater detail the degree to which 5XFAD mice mirror the progressive neuron loss observed in human AD, we counted neurons from female 5XFAD mice at ages 4, 6, 9, and 12 months by design-based unbiased stereology. Parasagittal 5XFAD and non-transgenic littermate control brain sections were stained with cresyl violet to visualize neuronal soma (Figure 1). At 4 and 6 months of age, no neuron loss in 5XFAD brain was apparent. However, by 9 months of age 5XFAD mice exhibited visible loss of large pyramidal neurons in cortical Layer 5 (Figure 1H) and subiculum (Figure 1Q), as previously reported [1]. In contrast, non-transgenic control mice showed no obvious neuron loss at any age. These results not only corroborate the 5XFAD neuron loss seen previously [10,16], but also support the notion of a progressive death of neurons that is absent at early ages.

**Figure 2:**
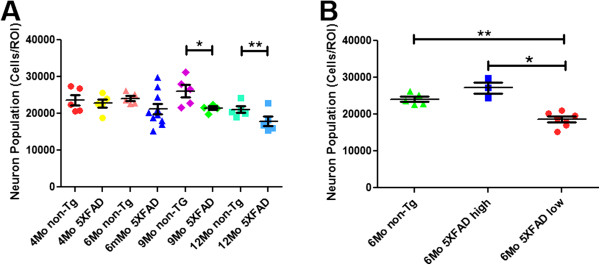
**Unbiased stereological analysis of 5XFAD mice reveals significant neuron loss in cortical Layer 5 at 9 and 12 months of age.** Brains from age-matched non-transgenic littermate (non-Tg) and 5XFAD female mice were sectioned at 30 μm and stained with cresyl violet and imaged by light microscopy. Large pyramidal neurons were then counted in Layer 5 cortex using an optical fractionator. **(A)** Large pyramidal neuron numbers in non-transgenic and 5XFAD Layer 5 cortex from mice aged 4–12 months. At 9 and 12 months there is a statistically significant decrease in the number of large pyramidal neurons in 5XFAD compared to non-transgenic mice (* p<0.05, ** p<0.01; n = 5 mice per group). 5XFAD mice exhibited a progressive decrease in Layer 5 large pyramidal neuron number, which at 12 months represented a 24.8% loss compared to non-transgenic mice. **(B)** 6 month 5XFAD mice are separable into two subgroups: those with normal neuron counts (6Mo 5XFAD high) and those with low neuron counts indicating neuron loss (6Mo 5XFAD low). The 5XFAD low count group showed a statistically significant decrease in neuron number compared to age matched non-transgenic and 5XFAD normal count groups (* p<0.05, ** p<0.01, n = 5 non-Tg, n = 10 net 6 month 5XFAD).

Next, we performed unbiased stereology to quantify the qualitative 5XFAD neuron loss that we observed. Quantitative analysis of large pyramidal neurons in Layer 5 of the cortex revealed a significant decrease in the number of neurons from 5XFAD mice at 9 and 12 months when compared to age matched non-transgenic littermate control mice (Figure 2A). At 6 months we observed a trend toward a decrease in neuron number within the 5XFAD group. Closer examination revealed two different 5XFAD mouse sub-groups: a normal group and another subset of mice that had succumbed to neuron loss (Figure 2B). The onset of neuron loss around 6 months of age appeared to be somewhat variable, but suggested that neuronal death may start in the 6 month old 5XFAD cohort. Neuron numbers of 5XFAD mice at younger ages and of all non-transgenic mice were not significantly different from each other. These results suggest a progressive death of neurons in 5XFAD mice that begins to become a major process around the age of 6 months and culminates in a ~25% loss of Layer 5 pyramidal neurons by 12 months of age.

Together, our results confirm and extend our previous qualitative work [10] and the quantitative study of Jawhar and colleagues [16] demonstrating neuron loss in the 5XFAD model. In addition, our data indicate that 5XFAD neuron loss occurs at earlier ages than previously shown and appears to begin when mice are about 6 months old. Thus, the 5XFAD model should prove useful for studies of Aβ-associated neurodegeneration and neuron loss.

### 5XFAD mice exhibit intraneuronal Aβ_42_ accumulation that precedes amyloid plaque formation

**Figure 3:**
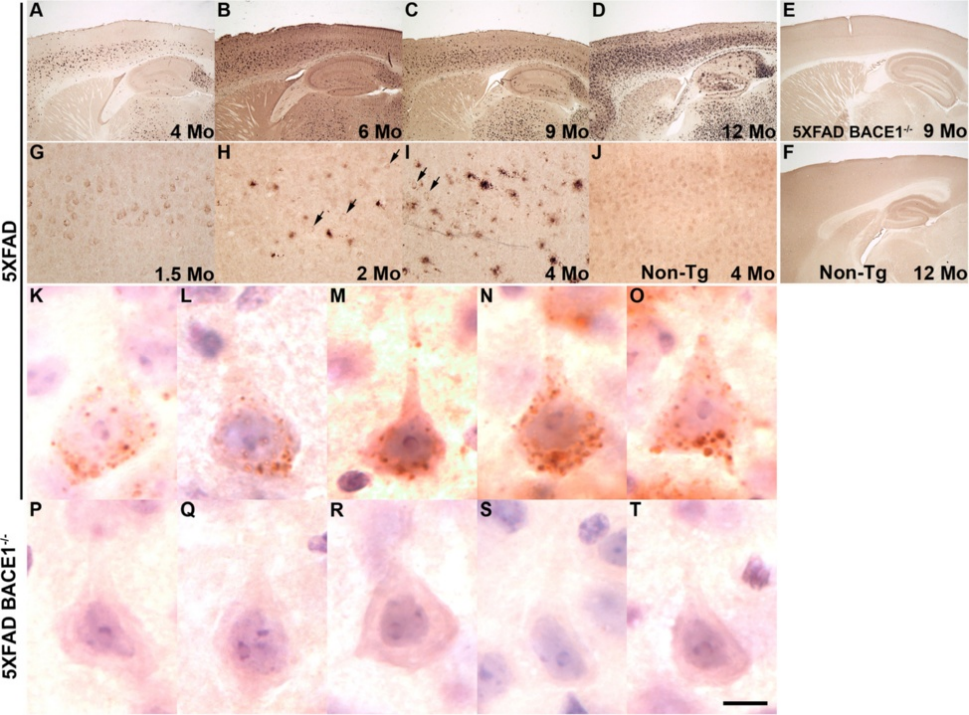
**5XFAD mice exhibit intraneuronal Aβ42 prior to amyloid deposition and neuron loss.** Parasagittal brain sections from 1.5 **(G)**, 2 **(H)**, 4 (**A, I-T**), 6 (**B**), 9 (**C**, **E**), and 12 (**D**, **F**) month mice were incubated with anti-Aβ_42_ C-terminal neo-epitope antibody, visualized by DAB staining, and micrographed. 5XFAD mice display progressive Aβ_42_-positive plaque deposition with age (**A-D**). In 5XFAD Layer 5 cortex, intraneuronal Aβ_42_ (arrows) is first observed at 1.5 months (**G**) and amyloid plaques at 2 months (**H**) of age. At high magnification, intraneuronal Aβ_42_ staining is confined within numerous small spherical puncta dispersed throughout the cytoplasm of the soma of large pyramidal neurons (**K-O**). The appearance of these Aβ_42_-positive puncta suggests membrane-bound intracellular compartments such as endosomes or lysosomes. Importantly, the absence of Aβ_42_ staining in 5XFAD; BACE1^−/−^ neurons (**E, P-T**) demonstrates the selectivity of the antibody for Aβ_42_, and indicates that the antibody dos not cross-react with endogenous or transgenic APP under the conditions used. Aβ_42_ staining is also lacking in non-transgenic littermate (Non-Tg) sections (**F**, **J**). Scale bar = 400 μm (**A-F**), 40 μm (**G-J**), 9 μm (**K-T**).

**Figure 4:**
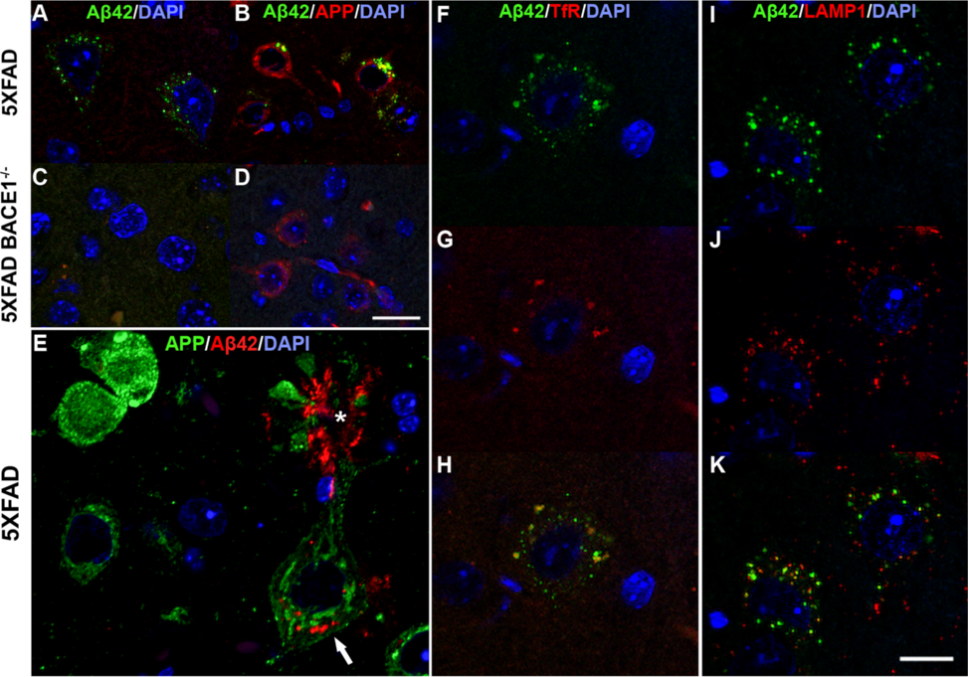
**Intraneuronal Aβ42 is partially localized within both endosomes and lysosomes in 5XFAD neurons.** Parasagittal brain sections of 5XFAD (**A**, **B, E-K**) and 5XFAD; BACE1^−/−^ (**C**, **D**) brains were co-stained with anti-Aβ_42_-selective antibody (**A-K**) and anti-APP (6E10: **B**, **D**; Karen [29]: **E**) or anti-Transferrin receptor (TfR) (**F-H**) or anti-LAMP1 (**I-K**) antibody, and DAPI for nuclei (**A-K**), and imaged by confocal microscopy. All mice were 4 months old, except mice used for LAMP1 immunostaining were 2 months (**I-K**). Label colors indicate fluorescence color. Note that APP staining appears widely distributed in the soma and cell surface (**B, D, E**), while Aβ_42_ labeling is confined to intraneuronal puncta in 5XFAD mice (**A, B, E, F, I**). Asterisk in **E** identifies Aβ_42_-positive plaque (red) above large pyramidal neuron that exhibits intraneuronal Aβ_42_ puncta (arrow). lntraneuronal Aβ_42_ signal is absent in 5XFAD; BACE1^−/−^ neurons (**C, D**), again demonstrating the Aβ_42_-selectivity of the antibody. In 5XFAD neurons, intraneuronal Aβ_42_ co-localizes with Transferrin receptor in early endosomes (**F-H**) and with LAMP1 in late endosomes and early lysosomes (**I-K**), at least in part. Scale bar in **D** = 12μm (**A, C**); = 20 μm (**B, D**). Scale bar in K = 12 μm (**E**); = 20 μm (**F-K**).

We anticipated that the 5XFAD neuron loss in cortical Layer 5 and subiculum would correspond to an elevated amyloid plaque load in these areas, as we previously observed [10]. To investigate this further, we performed immunohistochemistry using a rabbit monoclonal antibody that selectively recognizes the carboxy (C)-terminal neo-epitope of Aβ_42_ generated after γ-secretase cleavage; this antibody has minimal cross-reactivity with full-length APP and APP C-terminal fragments (CTFs; Figure 3 and 4). As expected, parasagittal 5XFAD brain sections incubated with the Aβ_42_ selective antibody displayed a progressive increase in amyloid burden with age (Figure 3A-D). At 4 months, 5XFAD mice exhibited moderate Aβ_42_ plaque deposition that was more dense in cortical Layer 5 and subiculum (Figure 3A). As 5XFAD mice aged through 6, 9, and 12 months, the number of Aβ_42_ plaques increased, and deposits began to accumulate in brain regions adjacent to Layer 5 and subiculum, such as CA1/2, CA3, dentate gyrus, Layers 4 and 6 of the cortex, and midbrain (Figure 3B-D). As expected, non-transgenic littermate control mice exhibited no Aβ_42_–positive deposits at any age (12 months, Figure 3F). As an additional negative control, we immunostained sections from a 9 month-old 5XFAD; BACE1^−/−^ mouse and observed no Aβ_42_ labeling (Figure 3E). Importantly, the lack of Aβ_42_ signal in 5XFAD; BACE1^−/−^ brain sections validated the selectivity of our anti-Aβ_42_ antibody, confirming that the antibody did not cross-react with full length APP or APP C-terminal fragments, and verified that plaque deposition requires BACE1 activity, as previously shown [17-20]. Together, these observations demonstrate that the regions with the greatest age-related Aβ_42_ deposition are also the same regions that exhibit neuron loss in 5XFAD brain.

### 5XFAD mice develop intraneuronal Aβ_42_ at an early age

Previous studies have suggested that intraneuronal Aβ is a potentially important factor participating in AD pathogenesis (reviewed in [8,21,22]). Our initial work demonstrated the presence of intraneuronal Aβ_42_ in 5XFAD mice starting at ~1.5 months [10], an observation that has been recently confirmed independently by several groups [10,16,23-26]. To verify intraneuronal Aβ_42_ in 5XFAD mice, we immunostained brain sections from 1.5, 2, and 4 month old mice with anti-Aβ_42_ selective antibody (Figure 3G-J). At 1.5 months, before the appearance of amyloid plaques, many large pyramidal neurons in cortical Layer 5 displayed numerous strongly stained Aβ_42_-positive puncta within their soma (Figure 3G). Intraneuronal Aβ_42_ staining was present when amyloid deposition began at ~2 months and persisted as plaque number increased with age (Figure 3H,I arrows). However, intraneuronal Aβ_42_ became more difficult to visualize over time, suggesting that either intraneuronal Aβ_42_ accumulation is transient [27], or intracellular Aβ_42_ signal is obscured as plaque staining intensifies with age. Non-transgenic littermate control sections lacked both plaques as well as intraneuronal Aβ_42_-positive puncta (Figure 3J), as expected.

The presence of intraneuronal Aβ in AD and APP transgenic brains has been controversial. One criticism against intraneuronal Aβ has been that anti-Aβ antibodies also recognize full-length APP and APP-CTFs, depending on the antibody (e.g., see [28]). To ensure that our selective anti-Aβ_42_ antibody was detecting bona fide intraneuronal Aβ_42_ and was not cross-reacting with APP or APP CTFs, we performed antibody titration experiments on both 5XFAD; BACE1^+/+^ and 5XFAD; BACE1^−/−^ brain sections. At a primary antibody dilution of 1:20,000, punctate Aβ_42_-positive staining was detected in the soma of large pyramidal neurons in cortical Layer 5 of 5XFAD; BACE1^+/+^ sections (Figure 3K-O), but no signal was observed in 5XFAD; BACE1^−/−^ sections (Figure 3P-T). Because 5XFAD; BACE1^−/−^ mice express the APP transgene but do not generate Aβ [18], these results strongly support the conclusion that our anti-Aβ_42_-selective antibody does indeed detect intraneuronal Aβ_42_ rather than full-length APP or APP CTFs.

To further confirm that our anti-Aβ_42_-selective antibody does not cross-react with APP, we performed confocal immunofluorescence microscopy on 5XFAD; BACE1^+/+^ and 5XFAD; BACE1^−/−^ brain sections co-stained with anti-Aβ_42_-selective and APP antibodies. 5XFAD sections showed punctate labeling of Aβ_42_ throughout the neuron soma (Figure 4A,B,E,F,I), while anti-human APP (6E10) staining revealed additional punctate and cell-surface labeling that were negative for Aβ42 (Figure 4B). As expected, Aβ_42_ signal was completely absent in the 5XFAD; BACE1^−/−^ sections (Figure 4C,D), but transgenic APP labeling was evident (Figure 4D), again demonstrating that our Aβ_42_-selective antibody does not recognize full-length APP or APP CTFs at the concentration used. Co-staining 5XFAD brain sections with anti-Aβ_42_ and a second anti-APP antibody (Karen, [29]) produced similar results (Figure 4E). Together, our data demonstrate that the anti-Aβ_42_ C-Terminal neo-epitope antibody does not have significant cross-reactivity with APP, and that punctate Aβ_42_ immunoreactivity resides within 5XFAD neuronal soma.

### Intraneuronal Aβ_42_ co-localizes with endosomal and lysosomal markers

The punctate Aβ_42_ immunolabeling in neuronal soma suggested that Aβ_42_ might be contained within membrane-bound subcellular compartments. Previous studies have indicated the presence of Aβ_42_ in endosomes and late endosomal multivesicular bodies in other models [30-32] and in cathepsin-D-positive puncta of 5XFAD mice [25]. To further investigate the subcellular localization of Aβ_42_ in 5XFAD neurons, brain sections were co-immunostained with anti-Aβ_42_ and anti-Transferrin receptor or anti-LAMP1 antibodies to identify endosomes or lysosomes, respectively, and imaged by immunofluorescence confocal microscopy. Indeed, we observed partial co-localization of punctate Aβ_42_ staining with signals from both Transferrin receptor and LAMP1 (Figure 4F-K). Although Aβ_42_ co-localization was observed with both markers, neither was exclusive. These results suggest the accumulation of intraneuronal Aβ_42_ throughout the endosomal-lysosomal system.

### 5XFAD brains exhibit increased Caspase-3 activation

**Figure 5:**
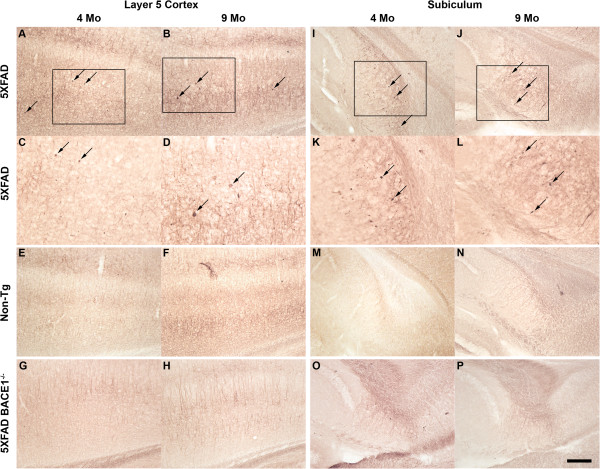
**5XFAD mice exhibit Caspase-3 activation in Layer 5 cortex and subiculum.** Parasagittal brain sections from representative 4 and 9 month old 5XFAD (**A-D, I-L**), non-transgenic littermate (Non-Tg; **E, F, M, N**), and 5XFAD; BACE1^−/−^ (**G, H, O, P**) mice were incubated with an antibody against activated Caspase-3, visualized by DAB staining, and micrographed in Layer 5 cortex (**A-H**) and subiculum (**I-P**). The levels of activated Caspase-3 immunolabeling are significantly higher in the brains of both ages of 5XFAD mice compared to those of 5XFAD; BACE1^−/−^ or non-transgenic mice. In addition, punctate aggregates of activated Caspase-3 are distributed throughout 5XFAD sections (black arrows, **A-D, I-L**), but are absent in 5XFAD; BACE1^−/−^ or Non-Tg sections (**E-H, M-P**). Boxes in **A, B, I,** and **J** are enlarged in **C**, **D, K,** and **L**, respectively. Scale bar **A, B**, **E-H, I, J, M-P** = 80 μm; **C, D, K, L** = 40 μm.

The pathogenic mechanisms leading to neuron loss in AD are not well understood. Studies in cultured human neurons have shown an association of intraneuronal Aβ_42_ with increased levels or activation of BAX and p53, suggesting that apoptosis may play a role in AD-related neuron loss [33]. To determine whether apoptosis might be involved in the loss of 5XFAD neurons, parasagittal brain sections were incubated with an antibody against the neo-epitope generated following activation of Caspase-3, a strong indicator of apoptosis. Overall, 5XFAD sections of Layer 5 cortex and subiculum displayed higher activated Caspase 3 immunostaining intensity than non-transgenic littermate and 5XFAD; BACE1^−/−^ sections (Figure 5A-D, I-L vs. 5E-H M-P). Close inspection of brain sections from 4 and 9 month-old 5XFAD mice revealed the presence of punctate aggregations of activated Caspase-3 (Figure 5A-D, I-L, arrows). These activated Caspase-3-positive puncta were smaller than neuron soma, typically ~2-8 μm in diameter, and were located almost exclusively in Layer 5 cortex and subiculum, the sites of localization of intraneuronal Aβ_42_ and 5XFAD neuron loss (Figure 5C,D,K,L, arrows). No Caspase-3-positive puncta were observed in non-transgenic littermate or 5XFAD; BACE1^−/−^ sections (Figure 5E-H, M-P). The presence of activated Caspse-3-positive accumulations was confirmed in 5XFAD Layer 5 cortex and subiculum at the ages of 6 and 12 months (data not shown), in addition to 4 and 9 months. Immunoblot analysis failed to detect the presence of activated Caspase-3 in 5XFAD whole-brain homogenates (not shown), however this was likely the result of dilution of the activated Caspase-3 signal below the level of detection. Overall, our results suggest that apoptotic processes are present in 5XFAD mice beginning at 4 months, before the start of neuronal death at 6 months, in the same brain regions that exhibit intraneuronal Aβ_42_, amyloid plaques, and neuron loss.

**Figure 6:**
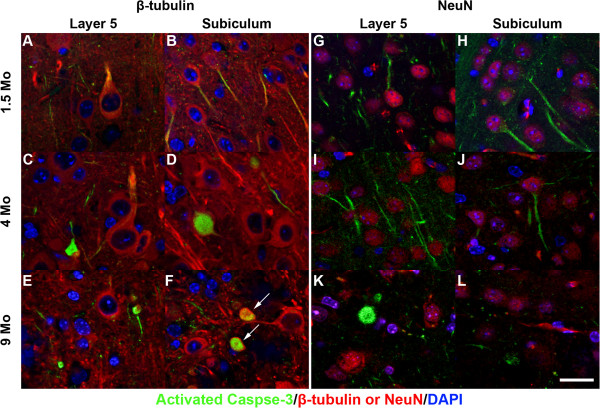
**5XFAD mice have elevated levels of activated Caspase-3 in neurons of Layer 5 cortex and subiculum.** Parasagittal brain sections from 1.5, 4, and 9 month old 5XFAD mice were co-incubated with antibodies against activated Caspase-3 (green) and neuron-specific class III β-tubulin (red; **A**-**F**) or NeuN (red; **G**-**L**) and imaged by confocal microscopy. Sections were co-stained with DAPI to detect nuclei (blue). Co-immunolabeling with neuronal markers β-tubulin and NeuN demonstrate Caspase-3 activation in 5XFAD neurons. Note the presence of activated Caspase-3 labeling in proximal dendrites of large pyramidal neurons of Layer 5 cortex and subiculum at 1.5 and 4 months (**A-D, G-J**), The number of Caspase-3-positive proximal dendrites is reduced in 9 month old compared to younger 5XFAD mice, although Caspase-3 aggregates are more prevalent at older ages suggesting a progressive process (spherical green structures in **E, F, K, L**). Activated Caspase-3-positive aggregates become apparent at 4 months in Layer 5 cortex and later in subiculum, and likely represent neuronal apoptotic bodies. Arrows in **F** point to Caspase-3-positive aggregates that co-label with neuron-specific class III β-tubulin. Scale bar = 20 μm.

The localization of activated Caspase-3 to regions of both Aβ_42_ accumulation and neuron loss implied potential causal links between these factors. To initially investigate this possibility, we first needed to confirm that activated Caspase-3 was indeed associated with neurons. Therefore, we co-incubated brain sections from 1.5, 4, and 9 month old 5XFAD mice with antibodies against the neuronal markers class III β-tubulin (TUJ1) or NeuN (A60) and activated Caspase-3, and performed immunofluorescence confocal microscopy. By 1.5 months, activated Caspase-3 immunostaining was identified in the soma and proximal dendrites of β-tubulin and NeuN-positive large pyramidal neurons in both Layer 5 cortex and subiculum (Figure 6A-D, G-J). At this age, activated Caspase-3 had not yet aggregated into puncta. However, by 4 months of age, spheroid accumulations of activated Caspase-3 had become apparent in Layer 5 cortex and subiculum (Figure 6C,D). In some cases, activated Caspase-3-positive puncta co-localized with β-tubulin in structures that were reminiscent of degenerating neurons (Figure 6F, arrows). Activated Caspase-3 co-localization with NeuN was less prominent, presumably because neuronal nuclei, the predominant site of NeuN subcellular localization, were largely degenerated (Figure 6K). These results confirmed the presence of activated Caspase-3 in 5XFAD neurons that were likely in the process of degeneration via apoptosis.

**Figure 7:**
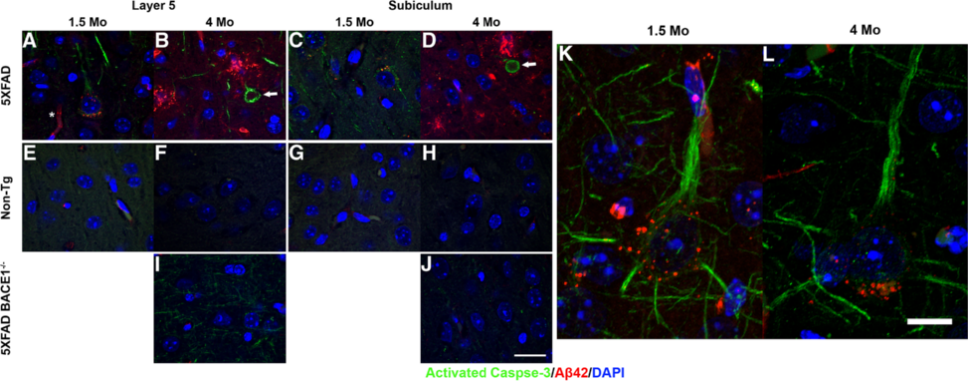
**5XFAD mice display Caspase-3 activation in neurons with intraneuronal Aβ42.** Parasagittal brain sections from 1.5 (**A, E, C, G, K**) and 4 month (**B, D, F, H-J, L**) 5XFAD (**A-D, K, L**), non-transgenic littermate (Non-Tg; **E-H**), and 5XFAD; BACE1^−/−^ (**I, J**) mice were co-incubated with antibodies against activated Caspase-3 (green) and Aβ_42_ C-terminal neo-epitope (red) and imaged by confocal microscopy. Sections were co-stained with DAPI to detect nuclei (blue). Activated Caspase-3 is present in 5XFAD large pyramidal neurons of Layer 5 cortex (**A, B, K, L**) and subiculum (**C, D**) that also display intraneuronal Aβ_42_. At high magnification, activated Caspase-3 is distributed throughout in the soma and proximal dendrites of Aβ_42_-positive neurons (**K, L**). Neither activated Caspase-3 nor intraneuronal Aβ_42_ are present in neurons of Non-Tg (**E-H**) or 5XFAD; BACE1^−/−^ (**I, J**) mice. Note presence of amyloid plaques (red) at 4 months in 5XFAD brain (**B, D**). Activated Caspase-3-positive ring-like structures (arrows, **B, D**) may represent neurons in the process of degeneration. Asterisk in A identifies background capillary staining. Scale bar **A-J** = 20 μm; **K, L** = 10 μm.

At this point, we had established the presence of activated Caspase-3 in large pyramidal neurons in Layer 5 cortex and subiculum of 5XFAD mice, but we had not demonstrated a direct association between activated Caspase-3 and intraneuronal Aβ_42_. Toward this end, we immunostained brain sections of 1.5 and 4 month old 5XFAD, non-transgenic littermate, and 5XFAD; BACE1^−/−^ mice with anti-activated Caspase-3 and anti-Aβ_42_-selective antibodies and imaged sections by confocal microscopy (Figure 7). Non-transgenic and 5XFAD; BACE1^−/−^ brain sections showed no specific immunostaining for Aβ_42_ or activated Caspase-3 (Figure 7E-J), confirming both the selectivity of the anti-Aβ_42_ antibody and the dependence of Caspase-3 activation on BACE1 cleavage of APP. In contrast, Layer 5 cortex and subiculum from 1.5 and 4 month old 5XFAD mice revealed both activated Caspase-3 and Aβ_42_ immunostaining (Figure 7A-D,K,L). Importantly, 5XFAD large pyramidal neurons that displayed activated Caspase-3 immunostaining in proximal dendrites, presumably representing an early stage of apoptosis, exhibited punctate intraneuronal Aβ_42_ labeling in soma (Figure 7K,L). 5XFAD neurons devoid of intraneuronal Aβ_42_ staining also lacked evidence of Caspase-3 activation. Interestingly, at 4 months of age, 5XFAD sections not only exhibited activated Caspse-3 positive aggregates in both the Layer 5 cortex and subiculum, but these profiles were consistently in close proximity to Aβ_42_-positive plaques (Figure 7B,D, arrows). Taken together, our results suggest apoptosis, via activation of Caspase-3, as a primary mechanism of neuron loss resulting from intraneuronal Aβ_42_.

## Discussion

Despite years of study, the mechanisms of neuron loss in AD are still incompletely understood. Apoptosis has been proposed as a potential mechanism leading to the neuronal death observed in this devastating neurodegenerative disorder (reviewed in [34-36]). Here, in the 5XFAD amyloid plaque mouse model, we have demonstrated progressive loss of neurons that correlates strongly with both intraneuronal Aβ_42_ and Caspase-3 activation. We observe a time course in 5XFAD mice in which intraneuronal Aβ_42_ first appears at 1.5 months of age in large pyramidal neurons of Layer 5 cortex and subiculum, concomitant with the observation of activated Caspase-3 in these neurons, but prior to amyloid deposition. By 4 months of age, 5XFAD mice exhibit amyloid plaques and the appearance of Caspase-3 positive puncta in Layer 5 cortex and subiculum that suggest neurodegeneration, but at a time when statistically significant neuron loss is not yet evident. At 6 months of age, 5XFAD mice as a whole show a trend toward neuron loss, in which Layer 5 neurons appear to split into two subgroups, those that display neuron loss and those that do not. However, by 9 months, neuron loss is statistically significant in Layer 5 cortex of 5XFAD mice. This time course, although correlative, suggests the possibility that intraneuronal Aβ_42_ triggers Caspase-3 activation, which in turn induces apoptosis-mediated neurodegeneration and eventual neuron loss in 5XFAD mice.

The 5XFAD mouse, along with the APP^SL^PS1KI, is one of a few known mouse models that exhibits significant neuron loss in addition to displaying other AD hallmarks such as amyloid plaques [10,11,16]. Our unbiased stereological counting of 5XFAD mice revealed a significant decline in large pyramidal neurons of the Layer 5 cortex starting at 9 months of age. Our data is consistent with previous studies, except we have demonstrated that significant 5XFAD neuron loss begins at an earlier age than previously reported [16]. Interestingly, a division develops at 6 months that appears to separate 5XFAD mice into those that exhibit significant neuron loss and those that have yet to surpass the point where neuron loss is occurring at a measurable rate. The ~6 month age period marks the first appearance of oligomeric N-terminally truncated Aβ peptides with pyroglutamate and an increasing decline of motor abilities in 5XFAD mice [16,23].

Consistent with previous studies [10,32], we observed intraneuronal Aβ_42_ as small puncta located in neuronal soma of 5XFAD mice. The intraneuronal Aβ_42_ became progressively more difficult to detect in 5XFAD sections after 1.5 months of age, presumably because highly concentrated Aβ_42_ plaques effectively blocked visualization of smaller and less prominent intraneuronal Aβ_42_ puncta. Despite this difficulty, 5XFAD mice exhibited a subset of neurons that harbored intraneuronal Aβ_42_ past 4 months. The early appearance of intraneuronal Aβ_42_ and its correlation with neuron loss in 5XFAD mice is also shared with the APP^SL^PS1KI transgenic mouse model [11,33,37,38].

We found that intraneuronal Aβ_42_ in 5XFAD mice was partially co-localized to endosomes and lysosomes, as indicated by co-labeling for Transferrin receptor and LAMP1, respectively. This corresponded well with previous findings that intraneuronal Aβ_42_ localizes to late endosomal multivesicular bodies [32] or lysosomes [25]; the latter observation is particularly significant, as it was obtained in the 5XFAD mouse. Intraneuronal Aβ_42_ localization to endosomes and lysosomes is also supported by previous studies that have proposed a mechanism in which the ubiquitin-proteasome system is disrupted, causing impaired protein degradation and increased Aβ accumulation [39,40].

It has been hypothesized that apoptosis is involved in the neuron loss seen in AD (reviewed in [34-36]). For example, activated Caspase-3 immunoreactivity has been reported to be present in AD brain [41,42] and in APP transgenic mice [43,44]. Interestingly, Aβ_42_ has been shown to cause cytochrome c release from mitochondria [45], which activates Caspase-3 and induces apoptosis, thus providing a potential mechanism for intraneuronal Aβ_42_-mediated neuron loss in 5XFAD mice.

In 5XFAD mice, activated Caspase-3-positive puncta were present throughout Layer 5 cortex and subiculum, often in close proximity to Aβ_42_-positive plaques. Caspase-3 activation was not apparent in both aged matched non-transgenic littermate and 5XFAD; BACE1^−/−^ mice. We speculate that these activated Caspase-3-positive puncta are likely the remnants of neurons that died by apoptosis, a conclusion supported by the observation of co-localized activated Caspase-3 and neuron-specific class III β-tubulin (Figure 6F). The Caspase-3 puncta in 5XFAD brain are reminiscent of activated Caspase-3-positive AD neurons that exhibit typical features of apoptosis such as condensed cytoplasm and shrunken nuclei [46,47]. In addition, the association of activated Caspase-3 and neuron loss in 5XFAD mice is consistent with the findings of apoptotic neuron death and “apoptotic bodies” found in a different PS/APP (PS1M146L and K670N/M671L) mouse model of AD [48]. While not always coexistent, we observed large pyramidal neurons from 1.5 month-old 5XFAD mice that contained both punctate intraneuronal Aβ_42_ within the neuron soma and activated Caspase-3 throughout the cell body and proximal dendrites (Figure 7K, L). We speculate that these neurons could represent an early stage of apoptotic neuronal degeneration and death. Interestingly, a recent study found activated caspase-3 in dendritic spines of hippocampal neurons in the Tg2576 APPswe transgenic mouse model [49], supporting the dendritic localization of activated Caspase-3 that we have observed in 5XFAD mice.

## Conclusions

The 5XFAD mouse has proven to be a useful model of amyloid deposition in the brain. Here, we have extended the utility of this transgenic mouse to the investigation of neuron loss that may have relevance to AD. We present compelling correlative data that associates intraneuronal Aβ_42_ with Caspase-3 activation and neuron death. Our data suggest a mechanism whereby intraneuronal Aβ_42_ accumulation in the endosomal-lysosomal pathway leads to activation of Caspase-3 and apoptotic neuron death in 5XFAD mice. Although some caution is warranted in interpreting results from an aggressive amyloid mouse model that overexpresses multiple FAD mutations, our results are consistent with evidence of activated Caspase-3 and apoptotic neuron death in human AD. Our study provides a framework that, with further elucidation of mechanisms, could lead to a deeper understanding of neuron loss in AD.

## Methods

### 5XFAD transgenic mice

5XFAD mice overexpress the K670N/M671L (Swedish), I716V (Florida), and V717I (London) mutations in human APP (695), as well as M146L and L286V mutations in human PS1. The generation of the 5XFAD mice has been described previously [10].

### Tissue collection

Mice were euthanized following IACUC approved procedures to minimize pain. After dissection, one hemibrain of each mouse was fixed in paraformaldehyde/PBS and cryo-preserved in 30% sucrose/PBS containing 0.01% sodium azide. Brains were sectioned sagittally on a freezing microtome at 30 μm. Serial sections were collected in 0.1 M phosphate buffer, pH 7.6.

### Stereology

Every sixth section was taken in sequential order, thoroughly washed in PBS, and then mounted on charged slides. Sections were allowed to dry between 1.5 to 2.5 hours. Slides were then taken through the cresyl violet staining procedure, where at each step the slides were slightly agitated every 15 seconds to ensure uniform staining. The sections were first briefly rinsed in water and then stained in 0.5% cresyl violet for 10 min. Sections were then rinsed in water briefly and then dehydrated in 50%, 70%, and 90% EtOH solutions for 30 seconds each. Sections were then de-stained in a 95% EtOH/1.4% glacial acetic acid solution for 1 min. Finally sections were dehydrated in a series of alcohols, cleared in xylene, and cover-slipped. The Layer 5 of the cortex was sampled for each section (1600 μm was examined starting at the point dorsal to the anterior most part of CA3 and then moving to the posterior of the brain). A Nikon (Tokyo, Japan) Eclipse 80i microscope with a motorized stage, StereoInvestigator 9 (Microbrightfield, Williston, VT, USA), and a 60x air lens was used to perform the stereological counting. Neuronal nuclei were then randomly sampled from the defined the Layer 5 using optical dissector probes, and a neuronal population was determined using a 2 μm top and bottom guard zone. Comparison of means using the *t*-test was performed using GraphPad Prism (GraphPad Software, Inc., San Diego, CA).

### Immunohistochemistry

Free-floating sections were washed in PBS with 0.4% Triton and then blocked in 3% goat serum for one hour. Sections were then incubated at 4°C on a shaker overnight with the following primary antibodies: rabbit monoclonal anti-Aβ_42_ (1:1000; Invitrogen; 700254) or rabbit monoclonal anti-cleaved Caspase-3 (1:1000; Cell Signaling; 9664). After more washes, the sections were incubated overnight at 4°C on a shaker with secondary biotinylated goat anti-rabbit at 1:1000. After a third set of washes, The Vector Laboratories (Burlingame, CA) ABC kit was used with 3,3′-Diaminobenzidine (DAB) as chromogen to visualize the reaction product. The sections were then mounted on charged slides, allowed to dry, dehydrated in a series of alcohols, cleared in xylene, and cover-slipped. Light microscopy was performed on a Nikon (Tokyo, Japan) Eclipse 80i microscope.

### Immunofluorescence confocal microscopy

Free-floating sections were treated with TBS with 0.1% SDS and 5 mM DTT for 30 min on a shaker. Sections were washed in TBS and then blocked in 5% goat or donkey serum for one hour (respective to the secondary being used). Sections were incubated at 4°C on a shaker overnight with the following primary antibodies: rabbit monoclonal anti-Aβ_42_ (1:1000; Invitrogen; 700254), mouse monoclonal anti-Aβ_42_ (1:1000; Covance; SIG-39142), rabbit monoclonal anti-cleaved Caspse-3 (1:1000; Cell Signaling; #9664), rat monoclonal anti-Transferrin receptor (1:500; Abcam; ab60344), rat monoclonal anti-LAMP1 (1:500; Abcam; ab25245), 6E10 mouse monoclonal anti-Aβ (1:1000; Chemicon; MAB1560), Karen goat polyclonal anti-N-terminal APP (1:500; [29]), DAPI (Invitrogen). After more washes, the sections were incubated for 1.5 h at room temperature on a shaker with Invitrogen Alexa Fluor secondary antibodies at 1:1000 (Donkey anti-Mouse or Rabbit-488 or 594; Goat anti-Mouse or Rabbit-488 or 594) and DAPI at 300nM. The sections were then mounted on charged slides and immediately cover-slipped using ProLong Gold (Invitrogen). Confocal images were captured on a Nikon (Tokyo, Japan) Eclipse C1si spectral laser scanning confocal microscope.

## Abbreviations

5XFAD: 5 familial AD mutations; Aβ: Amyloid-beta; AD: Alzheimer’s disease; CA: Cornu ammonis; CTF: Carboxy-terminal fragment; BACE1: β-site APP cleaving enzyme-1; LAMP-1: Lysosomal-associated membrane protein 1; APP: Amyloid precursor protein; BAX: Bcl-2-associated X protein; PS1: Presenilin 1; PS2: Presenilin 2; CTFs: C-terminal fragments; NeuN: Neuronal nuclei; TfR: Transferrin receptor.

## Competing interests

The authors declare that they have no competing interests.

## Authors’ contributions

WE performed experiments and wrote the manuscript. RV conceived experiments and edited the manuscript. Both authors read and approved the final manuscript.
